# Effects of Cortical Cooling on Activity Across Layers of the Rat Barrel Cortex

**DOI:** 10.3389/fnsys.2020.00052

**Published:** 2020-08-04

**Authors:** Gulshat Burkhanova, Kseniya Chernova, Roustem Khazipov, Maxim Sheroziya

**Affiliations:** ^1^Laboratory of Neurobiology, Kazan Federal University, Kazan, Russia; ^2^Aix Marseille University, INSERM, INMED, Marseille, France

**Keywords:** cortical cooling, slow waves, cooling-evoked desynchronization, multi-unit activity, sensory-evoked potential, barrel cortex, cortical layers, urethane

## Abstract

Moderate cortical cooling is known to suppress slow oscillations and to evoke persistent cortical activity. However, the cooling-induced changes in electrical activity across cortical layers remain largely unknown. Here, we performed multi-channel local field potential (LFP) and multi-unit activity (MUA) recordings with linear silicone probes through the layers of single cortical barrel columns in urethane-anesthetized rats under normothermia (38°C) and during local cortical surface cooling (30°C). During cortically generated slow oscillations, moderate cortical cooling decreased delta wave amplitude, delta-wave occurrence, the duration of silent states, and delta wave-locked MUA synchronization. Moderate cortical cooling increased total time spent in the active state and decreased total time spent in the silent state. Cooling-evoked changes in the MUA firing rate in cortical layer 5 (L5) varied from increase to decrease across animals, and the polarity of changes in L5 MUA correlated with changes in total time spent in the active state. The decrease in temperature reduced MUA firing rates in all other cortical layers. Sensory-evoked MUA responses also decreased during cooling through all cortical layers. The cooling-dependent slowdown was detected at the fast time-scale with a decreased frequency of sensory-evoked high-frequency oscillations (HFO). Thus, moderate cortical cooling suppresses slow oscillations and desynchronizes neuronal activity through all cortical layers, and is associated with reduced firing across all cortical layers except L5, where cooling induces variable and non-consistent changes in neuronal firing, which are common features of the transition from slow-wave synchronization to desynchronized activity in the barrel cortex.

## Introduction

Temperature strongly affects brain activity and metabolism, and vice versa (Kiyatkin, 2007; Wang et al., 2014). A cortical temperature below 32°C with normal core body temperature is one of the conditions for reporting brain death, and this is associated with a profound depression of EEG activity, although cortical activity *per se* may persist even at lower temperatures (Goila and Pawar, 2009; Wang et al., 2014). Therapeutic hypothermia is used for the treatment of hypoxic-ischemic encephalopathy in neonates (Gluckman et al., 2005; Shankaran et al., 2005; Thoresen and Whitelaw, 2005; Gunn et al., 2017; Lemyre and Chau, 2018) and for the prevention of hypoxic brain injury during cardiac arrest in cardiac surgery (Hypothermia after Cardiac Arrest Study Group, 2002; Andresen et al., 2015; Sekhon et al., 2017). Local cortical hypothermia is also used to control seizures (Motamedi et al., 2013) and has been investigated as a tool for the treatment of focal brain ischemia (Miyazawa et al., 2003; van der Worp et al., 2010). Monitoring of electrical cortical activity under these conditions is instrumental for an investigation into the brain’s survival and decision-making (Abend et al., 2011, 2013; Boylan et al., 2015; Frauscher et al., 2017). Hence the importance of understanding the effects of hypothermia on cortical activity under normal and pathological conditions.

Several neuronal and synaptic mechanisms of cooling have been found *in vitro*. First, a reduction in temperature to below 37°C decreases the activity of potassium leak channels and causes neuronal depolarization and an increase in input resistance (Volgushev et al., 2000; Trevelyan and Jack, 2002). Second, a moderate reduction in temperature diminishes synaptic vesicle release probability and/or desynchronizes neurotransmitter release (Katz and Miledi, 1965; Jasper et al., 1970; Hardingham and Larkman, 1998; Volgushev et al., 2004; Yang et al., 2005). Deep cooling induces strong depolarization, subsequent inactivation of fast sodium channels and depolarization block of the action potentials, and inhibits synaptic release (Jasper et al., 1970; Volgushev et al., 2000). Moderate cooling allows a change in the network balance in terms of neuronal membrane excitability (depolarization and increased input resistance) vs. synaptic excitability (reduced vesicle release probability and asynchronous release). The mechanistic similarity between moderate cortical cooling and the action of arousal neuromodulators was detected and it has been suggested that cooling can manipulate thalamocortical slow-wave activity *in vivo* (Sheroziya and Timofeev, 2015; Schwalm and Easton, 2016).

The cortical network displays bistable behavior during the synchronized states occurring during slow-wave sleep and general anesthesia, which can be detected as slow/delta waves in EEG associated with intermittent active (depolarized or UP) and silent (hyperpolarized or DOWN) states at the cellular level (Steriade et al., 1993, 2001; Lee and Dan, 2012; Neske, 2015; Poulet and Crochet, 2018). During wakefulness and rapid eye movement sleep, which are characterized by EEG desynchronization, neuromodulators such as acetylcholine partially suppress potassium channels and depolarize the membrane potential of cortical neurons, in particular, large L5 pyramidal cells (Krnjević et al., 1971; McCormick, 1992; Steriade et al., 1993; Hasselmo and McGaughy, 2004; Eggermann et al., 2014; Baker et al., 2018). In the cortex, acetylcholine is known to reduce the synaptic release and short-term synaptic depression (Gil et al., 1997; Hasselmo and McGaughy, 2004; Eggermann and Feldmeyer, 2009; Hasselmo and Sarter, 2011). Partial suppression of potassium leakage channels and a reduction of synaptic vesicle release probability, used to simulate the impact of neuromodulators *in silico*, transformed network slow-wave activity into persistent activation (Bazhenov et al., 2002; Compte et al., 2003; Hill and Tononi, 2005). Along with these findings, moderate cortical cooling eliminated silent states during slow oscillations in lightly anesthetized mice and prevented slow-wave generation in non-anesthetized head-restrained mice (Sheroziya and Timofeev, 2015). However, the changes in neuronal activity across cortical layers and sensory-evoked responses during cooling-evoked desynchronization remain largely unknown. It was shown previously that desynchronization increases or decreases neuronal firing in L5 while superficial layers display a more uniform decrease in firing during the transition from slow-wave to persistent activity (see “Discussion” section).

Here, using multi-channel recordings with linear probes from the barrel cortex, we found that the effects of cortical cooling on electrical activity are complex and differ across cortical layers, and involve an increase in the total time spent in active states with heterogeneous changes in L5 multi-unit activity (MUA) firing rates and reduced firing rates in all other cortical layers. The strongest decline in MUA rates was observed in the layer 4 (L4) and at the putative border between layers 5 and 6, which are the main recipient layers of thalamic sensory inputs. We proposed that hypothermia changes the excitation/inhibition balance in these layers. Overall our findings are in agreement with the hypothesis that the effects of moderate cooling on cortical network activity are associated with a transition of the cortex towards a state which resembles a desynchronized cortical state.

## Materials and Methods

### Ethical Approval

Experiments were performed following EU Directive 2010/63/EU for animal experiments, and the animal-use protocols were in line with Kazan Federal University on the use of laboratory animals (ethical approval by the Institutional Animal Care and Use Committee of Kazan State Medical University N9-2013).

### Surgery

Adult (1–2 months old) Wistar rats of both sexes were used. Surgery was performed under isoflurane anesthesia (4% for induction, 2% for maintenance), and urethane (0.7 g/kg, i.p.) was injected by the end of surgery. To reduce blood pressure and the possibility of bleeding, xylazine (10 mg/kg, i.p.) was injected at the beginning of surgery. Animals were placed on a warm thermal pad (37°C). The head was fixed in the stereotaxic frame and the skull of the animal was cleaned of skin. To reduce brain pressure and pulsation, we opened the fourth ventricle. A cranial window (~8 × 5 mm) was drilled out above the left hemisphere and the dura was gently dissected and removed above the barrel cortex (~1 × 0.5 mm). A chlorided silver wire placed in the cerebellum was used as the reference electrode. In the experiments with cortical perfusion, the craniotomy was encircled with a 2–3 mm-high dental cement wall to form a chamber.

### Cortical Cooling

To change the cortical temperature we used either perfusion with artificial cerebrospinal fluid or a U-shaped duralumin plate with a Peltier module (Sheroziya and Timofeev, 2015). We found that both methods were equally efficient. The cortex was perfused with ACSF at a speed of 6–8 ml/min. The ACSF temperature was maintained by a heater controller (Warner Instrument Corp., Hamden, CT, USA), at 38°C in control and 29–30°C during cooling. The ACSF temperature was monitored with a temperature sensor fixed inside the dental cement chamber. The duralumin plate was gently placed on the cortex and the craniotomy with the plate was covered by agar (4%) in saline. To monitor the temperature, the sensor was attached at the end of the duralumin plate by thermal conductive glue. The temperature of the plate was maintained with the Peltier element, at 38°C in control and 29–30°C during cooling. To reduce noise during the recordings the Peltier element was powered by a battery. The experiment lasted ~40 min and composed of three cooling sessions with recovery. The experiment (*n* = 22) was repeated twice in each animal (*n* = 11 rats) with a 1-h interval.

### Extracellular Recordings

LFPs and MUA were recorded from a barrel column using 16-site linear silicon probes with 100 μm distance between the neighboring recording sites (NeuroNexus Technologies, Ann Arbor, MI, USA). The probe was inserted into the cortex to a depth of 1,600–2,000 mm to cover all cortical layers with the recording sites. The extracellular signals from the probes were acquired using a Digital Lynx amplifier (Neuralynx, United States), digitized at 32 kHz, and saved for *post hoc* analysis. The whiskers were cut to a length of 4–5 mm and attached to a piezoelectric bending actuator (Noliac, Denmark). The principal whisker was identified by the shortest (compared to other whiskers) latency of MUA responses and was stimulated every 5 or 10 s by 200 ms long square pulse deflection in the caudal direction (amplitude of the deflection ~3 mm).

### Data Analysis and Statistics

Signals were analyzed using custom-developed MATLAB scripts (MathWorks, United States). For the spontaneous activity analysis, we excluded 1 s of the recordings after the whisker stimulus. For power analysis, we calculated delta (0.5–4 Hz), theta (4–7 Hz), alpha (7–15 Hz), beta (15–30 Hz), gamma (30–90 Hz), fast gamma (90–200 Hz) and “ultrafast” gamma (200–500 Hz) power within a 10 s sliding window.

**Figure 1:**
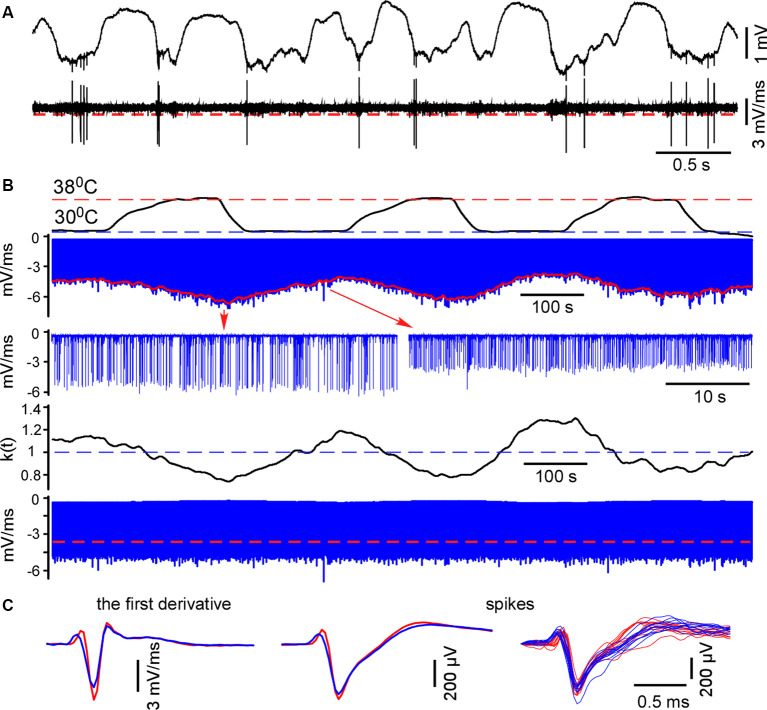
Spike detection during cortical cooling. **(A)** The upper trace represents the original wide-band signal recorded in L5. The lower trace is the first derivative of the original signal. **(B)** All negative peaks in the differentiated signal were detected (the upper blue trace, L5). To detect spikes in control and during cooling the envelope was plotted (shown with red). The correcting coefficient k(t) was calculated and the negative peaks were multiplied by k(t). Finally, spikes were detected using constant threshold = 70% of the mean maximal amplitude (the lower trace, shown with a red dashed line). **(C)** Examples of the averaged spike first derivative, averaged spikes and superposition of spikes are shown in control and during cooling (38°C—shown with red, 30°C—blue).

For spike detection, we differentiated the raw wide-band signal and located negative peaks (minimal slopes). Extracellular spike amplitudes and slopes interdependently decline during cooling (Henze et al., 2000; Volgushev et al., 2000). To compare MUA/spike firing rates and plotting spike histograms in control and during cooling we plotted the envelope (Figure 1) and calculated the correcting coefficient k(t) = mean[envelope]/envelope(t). Negative peaks were multiplied by the correcting coefficient k(t). Then we redetected negative peaks with the constant threshold = 70% of the mean maximum value. Extracellular events with slopes below ||−1|| mV/ms were discarded. Detected spikes were inspected and averaged. Mean firing rates were calculated for control and hypothermic conditions. We detected spontaneous and sensory-evoked spikes independently because, as a rule, they had different amplitudes. To detect sensory-evoked spikes we consistently collected 40 ms epochs of the recordings after the stimulus and applied the same correction method as above. The MUA grouping index for spontaneous activity was calculated as the mean number of spikes within the ±10 ms time window around a spike.

Similarly to spikes, cortical cooling decreased slow-wave amplitude (Figure 3), and using a constant threshold for slow-wave detection might mislead. To analyze changes in slow-wave activity in detail we used “noisy spikes” [all negative peaks above ||−1|| mV/ms after differentiation of the raw wide-band local field potential (LFP) signal] in L5 and calculated interspike intervals (ISIs; Figure 4A). For each ISI we detected the maximal L5 LFP value between the pair of spikes and L5 LFP amplitudes corresponding to each spike (Figure 4A). Plotting the indicated factors against each other, 2 clusters representing active and silent states were detected (Figure 4B). For automatic cluster discrimination, k-means clustering was used. Time spent in silent and active states was calculated as a sum of “silent” and “active” ISIs, respectively. The slow-wave frequency was calculated as the number of “silent” ISIs per second. Near the end of each “silent” ISI, the minimal value of the LFP (band-pass 0.5–6 Hz) slope was detected and used for averaging delta waves (zero time in Figure 5A). To calculate a depth profile for slow-wave onset (vertical propagation) and termination, the LFP signals from the recording channels were normalized to the maximal amplitude after the onset and before the silent state respectively (Figure 5E). Then, using threshold 0.1 for onsets and 0.5 for termination we detected threshold-crossing times for each channel and used L5 as a reference channel (zero time, Figures 5E,F). To analyze the slow-wave associated MUA synchrony we combined spikes from all the recording channels and plotted spike histograms. The spike histogram slopes were calculated as maximal values of the first derivatives for ascending phases of the MUA response. Sensory evoked potential (SEP) and slow-wave slopes were calculated as the minimal values of the first derivative (Figures 5, 6).

**Figure 2:**
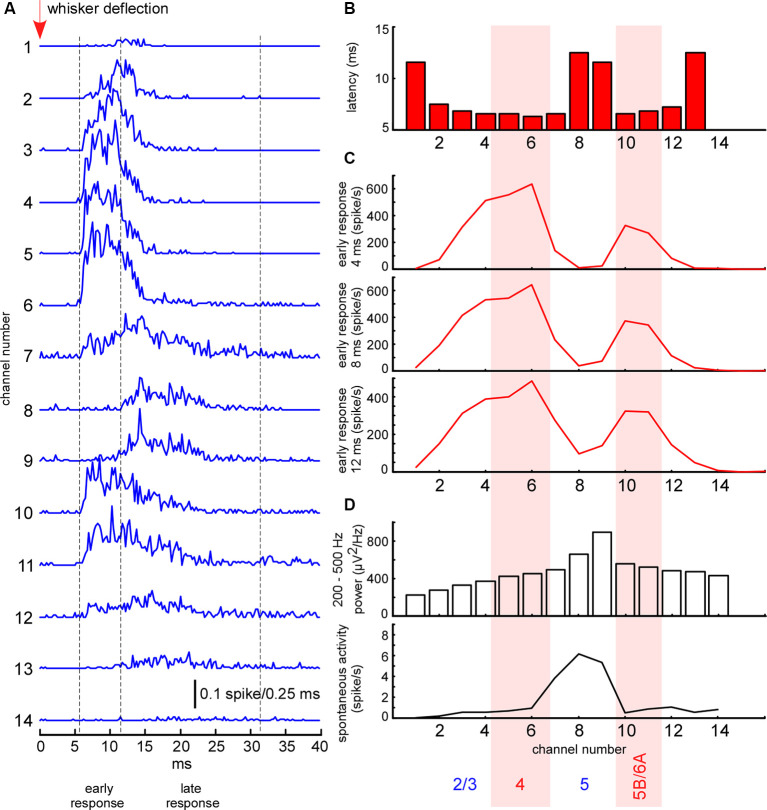
Electrophysiological clues for layer detection. **(A)** Averaged spike histograms for each channel (the last two channels not shown) represent averaged sensory-evoked multi-unit activity (MUA) response in the barrel cortex to the principal whisker stimulation. **(B)** The latency of sensory-evoked MUA response shown in panel **(A)** calculated for each recording channel. **(C)** Mean MUA firing rate of the response during the first 4, 8, and 12 ms after the response onset. **(D)** Fast gamma (200–500 Hz) power and spontaneous MUA firing rate for each recording channel.

**Figure 3:**
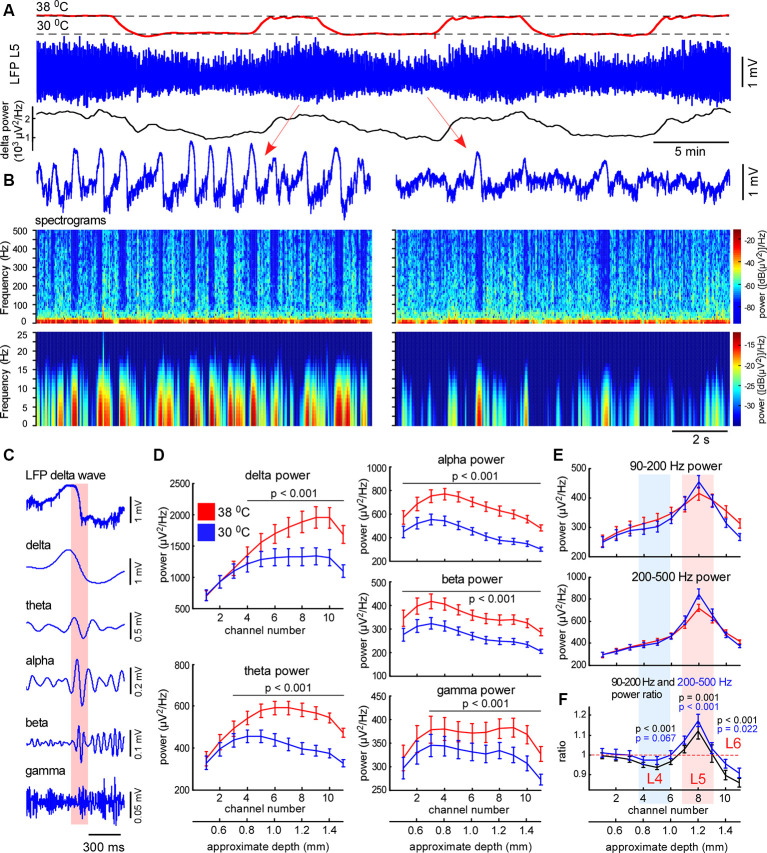
Moderate cortical cooling decreased slow-wave activity and increased fast gamma power in L5. **(A)** Moderate cortical cooling reversibly decreased the frequency and amplitude of the slow waves. The red, blue, and black traces represent the cortical surface temperature (38°C—control, 30°C—cooling), the L5 local field potential (LFP) signal, and delta power, respectively. **(B)** Examples of L5 LFP slow-wave activity with spectrograms in control and during cooling. **(C)** Example of an original L5 LFP signal (one delta wave is shown) and the corresponding band-passed signals within the indicated frequency ranges. In the shown example, the transition from the silent to the active state (highlighted with pink) contributes to theta, alpha, and beta power. **(D)** Averaged delta, theta, alpha, beta and gamma power for each recording channel of the silicon probe in control and during cooling (*n* = 22). Activities were averaged around the fast gamma (200–500 Hz) peak in L5 (reference channel, indicated as channel 8). **(E)** Fast gamma power (90–200 Hz and 200–500 Hz) averaged around the fast gamma peak in L5 (reference channel, indicated as channel 8). **(F)** The ratio of fast gamma power (cooling/control) was significant >1 in L5 and <1 in L4 and 6. The 90–200 Hz power and 200–500 Hz power ratio are shown with black and blue respectively. *P-values* are shown for channel 5 (L4), 8 (L5), and 10 (L6).

**Figure 4:**
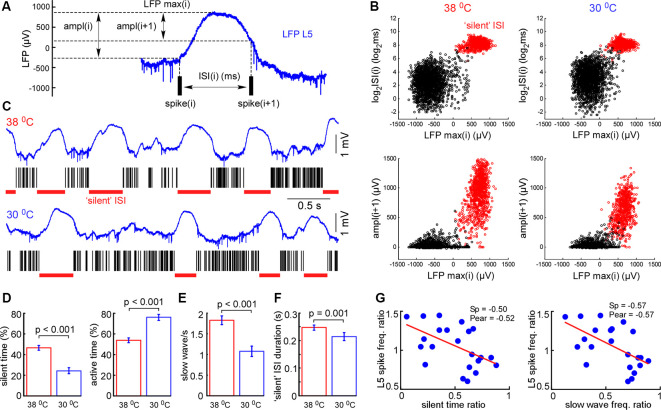
Cortical cooling decreased slow-wave occurrence and total time spent in silent states. **(A)** Schematic representation of the analysis in L5. For each interspike interval (ISI) we calculated its duration, maximal LFP value between the spikes, and LFP amplitudes [ampl(i) and ampl(i + 1)]. **(B)** Plotting the indicated factors against each other, two clusters corresponding to active and silent states were detected. For automatic cluster discrimination, we used k-means clustering. **(C)** Examples of L5 LFP signals with discriminated ISIs in control and during cortical cooling. **(D)** The mean time spent in silent (calculated as a sum of all “silent” ISIs) and active states in control and during cortical cooling (*n* = 22). **(E)** Slow-wave occurrence in control and during cooling. **(F)** Mean duration of silent states (“silent” ISI) in control and during cooling. **(G)** Ratio (cooling/control) of spike frequency in L5 plotted against silent time ratio and slow-wave occurrence ratio (one dot represents one experiment, *n* = 22). The stronger silent time and slow-wave occurrence were decreased with cooling, the higher L5 spike frequency was increased. Linear fitting is shown with a red line. Spearman and Pearson correlation coefficient is indicated as Sp and Pear, respectively.

**Figure 5:**
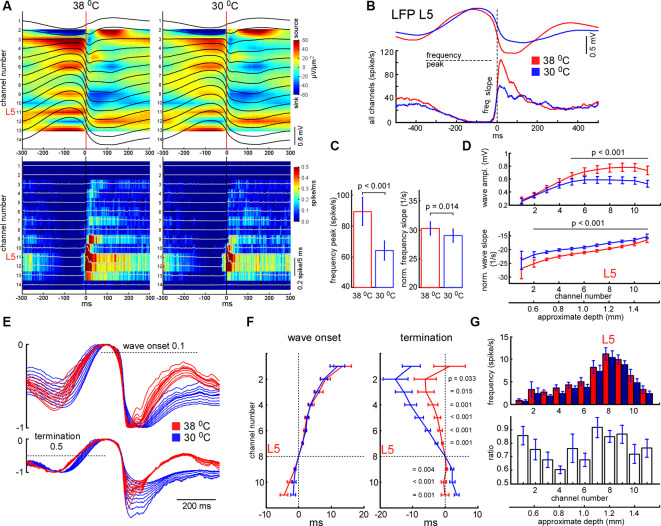
Effects of cooling on slow-wave activity and associated spike synchrony. **(A)** Example of averaged LFP slow-wave with current source density plot in control and during cortical cooling (upper plots) and the corresponding MUA histograms and density plots (bottom). Activities averaged around (triggered by) the minimal value of the L5 LFP slope (zero time). **(B)** Example of averaged L5 LFP slow waves and spike histograms in control (shown with red) and during cortical cooling (blue). Here spikes were collected from all the recording channels of the silicon probe. **(C)** Cortical cooling significantly decreased the delta wave associated spike frequency peak (shown in **B**) and its slope during the transition from silent to the active state (*n* = 22). **(D)** Averaged slow wave amplitudes and the wave onset slopes calculated for each recording channel in control and during cooling. Activities were averaged around L5 (channel 8). **(E)** The imposition of normalized slow-wave signals recorded with the silicon probe through the cortex during cooling. **(F)** Vertical propagation of slow waves in control and during cooling. Cooling did not change the depth profile calculated for the wave onset (threshold 0.1); on average, slow waves started in the deep layers and propagated upward. Cooling significantly changed the depth profile calculated for the wave termination (threshold 0.5); despite the pattern being different in each experiment, signals from the superficial layers crossed the threshold earlier with cooling. *P* values calculated for cooling-evoked increments < and >0 for channels 1–7 and 9–11, respectively (*n* = 22). Activities were averaged around L5 (zero time). **(G)** Mean firing rates and their ratios during the first 100 ms after the slow-wave onset (*n* = 22).

**Figure 6:**
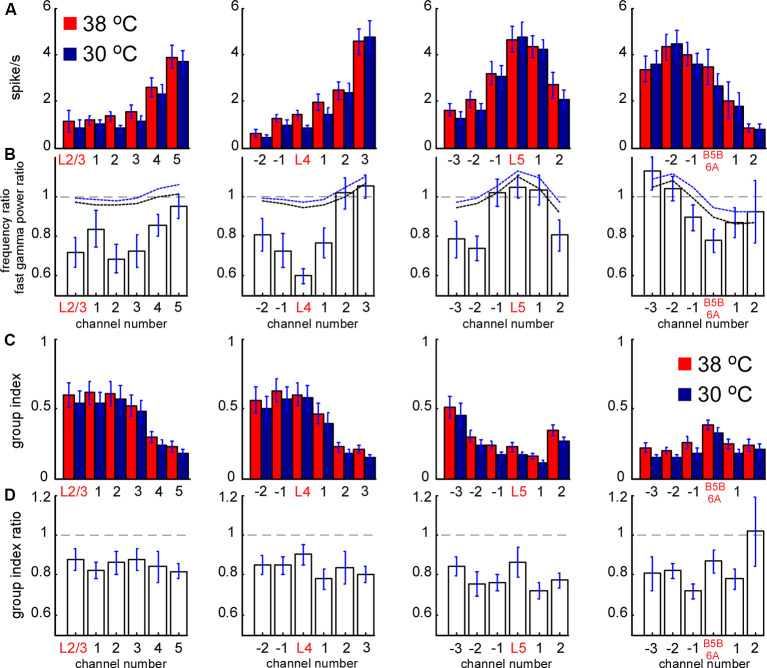
Spontaneous MUA rate in control and during cortical cooling. **(A)** MUA firing rates from different channels of the silicon probe averaged around cortical layers (representative channels) in control (shown with red) and during cortical cooling (blue color). **(B)** Bars represent the MUA frequency ratio (cooling/control) averaged around cortical layers. Dashed black and blue lines represent averaged fast gamma power of the 90–200 Hz and 200–500 Hz ratio, respectively (*n* = 22). **(C)** MUA grouping index calculated for different cortical layers in control and during cooling. The grouping index was calculated as a mean number of spikes in a ±10 ms time window around a spike. **(D)** MUA grouping index ratio averaged around cortical layers in control and during cooling.

The whisker-evoked response in the barrel cortex of anesthetized rats (triphasic response) is composed of fast initial excitation, a prolonged inhibition and then delayed excitation (Chapin et al., 1981; Armstrong-James and George, 1988; Zhu and Connors, 1999). We analyzed the sensory-evoked MUA response corresponding to the fast initial excitation, divided into early and late components. In all experiments, the early short-latency MUA response revealed two separate peaks of maximal firing rates, located in layer 4 and at the border between layers 5 and 6 (Figure 2B). The duration of the early response was defined individually in each experiment, starting from the response onset, and, so long as firing rates maintained the same depth profile, composed of two peaks (7–12 ms, Figure 2B). The late evoked MUA response was calculated within the 20 ms time window after the early component. To detect evoked MUA response latency we plotted the averaged spikes histogram and detected the moment they reached 25% of the maximal value during the sensory response.

To detect cortical layers we used known electrophysiological clues (Figure 2). L5 thick-tufted pyramidal cells display the highest spontaneous firing rate in the rat primary somatosensory cortex (de Kock et al., 2007; Reyes-Puerta et al., 2015; Fiáth et al., 2016). As compared to other cortical layers, L5 also displays the highest “spike power” (Senzai et al., 2019). For this reason, L5 (representative channel) was detected by the peak in fast gamma power (priority) and by the highest spontaneous MUA firing rate. All types of excitatory neurons in the rat barrel cortex potentially receive direct thalamocortical inputs, but cortical neuron density and thalamocortical VPM bouton density are highest in L4 and approximately at the border between the layer 5B and 6A (Meyer et al., 2010a,b; Constantinople and Bruno, 2013; Vinokurova et al., 2018), so it is logical to expect high MUA density during early short-latency sensory-evoked response within these layers. L4 and the putative border of 5B/6A (B5B/6A, representative channels) were detected by the frequency peaks in the early sensory-evoked response. L2/3 (representative channel) was formally detected 200–300 μm above layer 4. In several experiments, we confirmed the validity of our layer detection methods with histology (not shown).

Statistical analysis was performed using the Matlab Statistics toolbox and OriginPro (OriginLab Corporation, Northampton, MA, USA). Data are expressed as mean ± SEM. If the normality and equal variance tests were passed, the two-tailed *t*-test was used; otherwise, the nonparametric Wilcoxon signed-rank test was performed (significance level, *p* < 0.05). To detect linear relations between variables we employed robust regression and calculated Spearman and Pearson coefficients.

## Results

### Moderate Cortical Cooling Decreases Slow-Wave Activity and Promotes Continuity of Neuronal Network Activity

To explore cooling effects on the slow-wave activity we recorded LFP and MUA with linear probes in normothermia and during moderate cooling in the rat barrel cortex. A drop in temperature reversibly decreased slow-wave activity (Figure 3A), that decrease was accompanied by reduced silent state occurrence, as revealed with spectrograms (Figure 3B). Layer-specific power analysis showed significant decreases in the delta, theta, alpha, beta, and gamma power during cooling through all layers (Figure 3D). It is important to note here, that fast transitions from silence to activity during delta wave generation strongly contribute to a broad range of frequencies (Figure 3C), thus, cooling-evoked suppression of slow-wave activity was followed by a drop in theta, alpha, and beta power. Cortical cooling significantly increased fast gamma power in L5, but significantly reduced it in L4 and 6 (Figures 3E,F).

Next, we analyzed changes in slow-wave activity in more detail (Figure 4). With this aim, we performed k-means clustering for ISIs and LFP activity in L5 (see “Materials and Methods” section). Slow waves are an alternation of the active (depth-negative phase in L5 LFP) and silent (depth-positive phase in L5 LFP) states. Two clusters, composed of short (and negative LFP value) and longer-duration (with positive LFP) ISIs (Figure 4B), represented the active and silent phases of cortical delta waves, respectively. The sum of the corresponding ISIs forming two clusters allowed us to estimate total time spent in the active and silent states. We found that cooling significantly decreased total silent time, and increased total active time, as well as decreased slow-wave occurrence (Figures 4D,E). Cooling lightly, but significantly decreased the duration of silent states estimated as the duration of “silent” ISIs (Figure 4F). Accordingly, the strongest decrease in silent time and slow-wave occurrence was associated with the strongest increase in L5 MUA mean firing (*n* = 22, Figure 4G), though, the grand average for L5 MUA rate was not changed by cooling (see below). Averaged delta waves under normothermic and hypothermic conditions are shown in Figure 5. We found that cooling significantly reduced delta wave amplitude and MUA modulation (synchronization) by delta waves (Figures 5B,G). This manifested as a decrease in peak MUA following the onset of the active state and a total decrease in firing during the 100 ms after the onset. Cooling significantly reduced delta wave amplitudes and the slopes of LFP delta waves, and MUA histograms triggered by the wave onsets (Figures 5B–D). Interestingly, we did not detect significant changes in vertical propagation of wave onsets during cooling (Figure 5F); on average, slow waves started in the deep layers and propagated upward (Sanchez-Vives and McCormick, 2000; Sakata and Harris, 2009; Chauvette et al., 2010; Reyes-Puerta et al., 2016). In contrast, cooling significantly changed the propagation profile calculated for the wave termination; the profile displayed high variability in different experiments (not shown), but the slow-wave termination in upper layers occurred earlier with cooling, so that, on average we detected propagation from superficial to deep layers. Taken together, these findings indicate that moderate cooling strongly depresses delta-wave activity through a decrease in delta-wave occurrence and promotes continuity (or desynchronization) of neuronal network activity.

### MUA Firing in Control and During Cortical Cooling

To reveal layer-specific changes in neuronal firing associated with a cooling-dependent decrease in slow-wave activity we analyzed MUA (Figure 1). Extracellular spike amplitudes and slopes decline during cooling and using a constant threshold for MUA detection might mislead. To detect MUA in control and hypothermia we corrected spike amplitudes and then used a constant threshold for detection (see “Materials and Methods” section) and calculated time average MUA firing rates for each cortical layer (Figure 6). To avoid potential variability of cortical size between different animals we averaged MUA obtained from each recording channel of the silicon probe around the detected representative channels in L2/3, L4, L5, and at B5B/6A (Figure 6A). Absolute values of MUA can be different in different experiments; therefore, to reveal relative changes, we plotted ratios (cooling/control) of MUA rates (Figure 6B) and ratios for fast gamma power (same as in Figure 3D, shown with black and blue dashed lines, Figure 6B). We found that moderate cortical cooling significantly decreased spontaneous firing rate in superficial, L4 and B5B/6A (*p* < 0.001, *n* = 22), but did not change it in L5. MUA rates in L5 showed heterogeneous changes during cooling, with some animals showing increased or decreased firing (Figure 4G). The total firing rate (sum of all recording channels) was significantly diminished by cooling (89 ± 3% of the control value, *p* < 0.001, *n* = 22). Qualitatively, the depth profile of the fast gamma power ratio was similar to the depth profile of the MUA ratio, with the maximum in L5 and minimum in L4 and around B5B/6A (Figure 6B). To estimate MUA grouping (synchrony) for each cortical layer we calculated the mean number of MUA spikes within a ±10 ms window around a spike (MUA grouping index) in control and during cortical cooling, and the ratio of cooling grouping index to control grouping index (Figures 6C,D). We found that the MUA grouping index was maximal in the superficial layers and L4 and had a local maximum at B5B/6A. Moderate cortical cooling diminished the MUA grouping index through all cortical layers. Thus, layer-specific analysis of MUA during cooling-evoked desynchronization revealed that the most significant decrease in firing rate is in the superficial layers, L4, and at B5B/6A.

### Cortical Sensory-Evoked Responses During Active and Silent States in Control and Cooling Conditions

**Figure 7:**
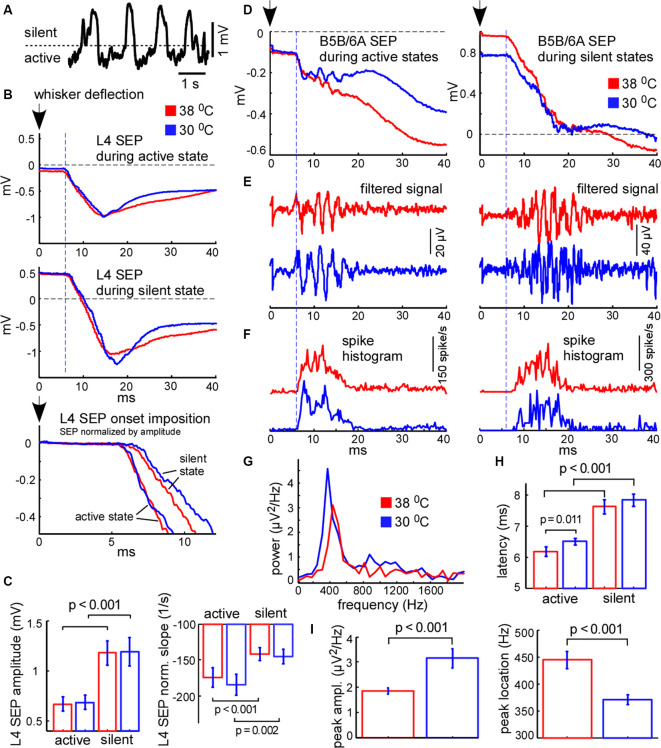
Cortical sensory-evoked response in control and during cooling: active state vs. silent state. **(A)** Example of slow-wave activity in L5. The dashed line represents the threshold (0.4 standard deviations of the LFP signal), used for sensory stimuli sorting. **(B)** Examples of averaged L4 sensory-evoked potentials (SEPs) evoked during the active and silent states in control (shown with red) and cooling conditions (blue). The black arrow (zero time) represents the moment of principal whisker deflection. The lower trace represents a superposition of sensory evoked potential (SEPs) normalized by amplitude. **(C)** L4 SEP amplitude was significantly higher if evoked during the silent state compared to the active state independently of temperature (*n* = 22). The L4 SEP slope normalized by amplitude was significantly higher if evoked during active states. **(D)** Examples of averaged B5B/6A SEPs evoked during active states (left panel) and silent states (right panel) in control and cooling conditions. The original wide-band signal was averaged. Note high-frequency oscillation. **(E)** The same activities as in **(D)** band-passed within the 300–16,000 Hz range. Note sensory-evoked high-frequency oscillation. **(F)** Spike histograms (spikes collected from all recording channels) in control and during cooling. Note the higher latency of the sensory-evoked MUA response during the silent state compared to the active state. **(G)** The power spectrum plot calculated for the activities shown in **(E)** for active states. The power spectrum plot revealed a high-frequency oscillation peak. Note, that cortical cooling increased the peak amplitude and decreased the frequency of high-frequency oscillation. **(H)** The latencies of sensory-evoked MUA responses were significantly higher if evoked from silent states compared to active states. **(I)** Cortical cooling significantly increased the power of sensory-evoked high-frequency oscillation (peak amplitude) and decreased its frequency (peak location).

We further compared cortical sensory responses evoked by the principal whisker deflection in control and cooling conditions. Since cortical sensory-evoked potentials (SEPs) depend on the phase of slow oscillation and the network state (see below) we analyzed the responses evoked during the active and silent cortical states separately. To sort responses evoked during different states we applied a threshold to the L5 LFP signal (Figure 7A). Because L4 SEP is known to be the largest compared to the other layers, we firstly calculated its amplitude and slope. We found that the amplitude of the L4 SEP was significantly higher if the principal whisker was deflected during the silent state as compared to the active state (Figures 7B,C) and did not depend on moderate cooling. The L4 SEP onset slope, normalized by amplitude, was significantly lower if the principal whisker was deflected during the silent state (Figure 7C) and also did not depend on moderate cortical cooling. To compare latencies of the sensory-evoked response we plotted spike histograms (Figure 7F). The latencies were significantly longer if the response was evoked during the silent state independently of moderate changes in temperature (Figure 7H). The latency during active state/hypothermia was significantly longer compared to the latency during active state/normothermia (6.5 ± 0.1 vs. 6.17 ± 0.15 ms respectively; *p* = 0.011, *n* = 22). We conclude that the amplitude, slope, and latency of the sensory-evoked response were much more dependent on the network state than on a moderate change in temperature.

It was previously shown that the sensory-evoked high-frequency oscillations (HFO) in the barrel cortex were eliminated with deep cooling that suppresses neuronal activity (Jones and Barth, 1999; Staba et al., 2003). To reveal moderate cooling effects on HFO we averaged LFP signals applied during active or silent states (Figure 7D) and band-passed them within the 300–16,000 Hz range (Figure 7E). We found that HFO was sensitive to moderate cortical cooling. A moderate drop in temperature significantly decreased HFO frequency (Figure 7I) but increased its peak power (Figures 7E,G,I). Also, HFO were generated later if the stimulus was applied during the silent state (Figure 7E).

It is known that the amplitude of the cortical response is higher during the silent (hyperpolarized) state as compared to the active (depolarized) state (Sachdev et al., 2004; Crochet et al., 2005), because stimulation during the silent state synchronously depolarizes and activates all the cells in the target area. The latency of cortical response during the silent state also appears to be longer because membrane potential transition from a hyperpolarized state to the threshold of spike generation takes more time (Shu et al., 2003a; Rosanova and Timofeev, 2005). Therefore, the relative response latencies and amplitudes obtained from extracellular recordings allowed us to make a judgment about the instantaneous membrane potential at the moment of sensory stimulation. Taken together, our results support the idea that cortical cells are depolarized during cooling-evoked active states which we detected with LFP signal and, therefore, active states under moderate hypothermia are mechanistically close to the true active states under normal physiological conditions.

### Sensory-Evoked MUA Firing During Cortical Cooling

**Figure 8:**
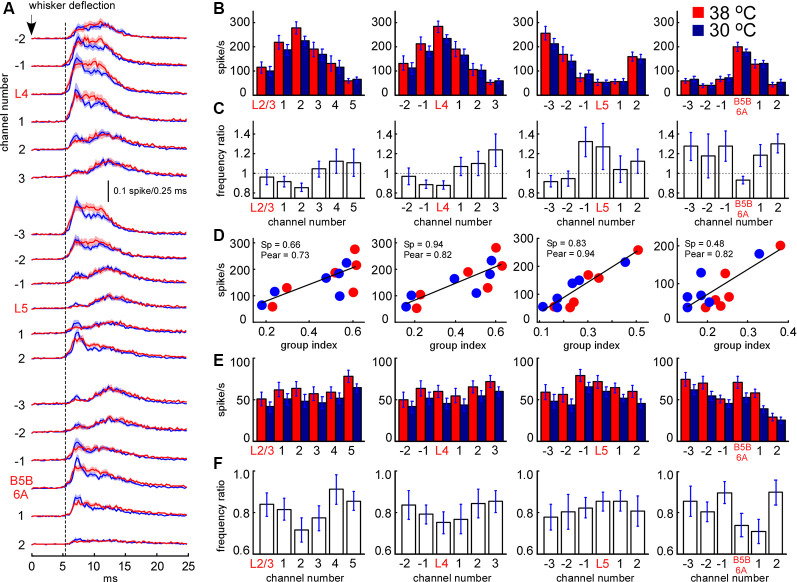
Sensory-evoked MUA rate in control and during cortical cooling. **(A)** Grand average peristimulus spike histograms (evoked during active states) in control (red) and during cortical cooling (blue). Note the increased peak firing with cooling. **(B)** Early sensory-evoked MUA firing rate (evoked during active states) from different recording channels of the silicon probe in control (shown with red) and during cortical cooling (blue). **(C)** Early sensory-evoked MUA frequency ratio (cooling/control) averaged around cortical layers (*n* = 22). **(D)** Early sensory-evoked MUA firing rate plotted against the MUA grouping index shown in Figure 7C. Cortical layers with a high grouping index (spontaneous activity) responded to the whisker stimulation with higher firing rates. Linear fitting is shown with black lines. Spearman and Pearson correlation coefficient is indicated as Sp and Pear respectively. **(E)** Late sensory-evoked MUA firing rate (evoked during active states) from different recording channels of the silicon probe averaged around cortical layers (representative channels) in control and during cortical cooling. **(F)** Late sensory-evoked MUA frequency ratio (cooling/control) averaged around the detected cortical layers.

We further analyzed the effect of cooling on MUA evoked by principal whisker deflection. MUA response was divided into an early (7–12 ms after the onset) and late component (the following 20 ms). As above, we plotted MUA rates for early (Figure 8B) and late components (Figure 8E) around the detected representative channels in L2/3, L4, L5, and at B5B/6A. We analyzed MUA responses evoked during active states (responses evoked during silent states revealed rather similar cooling-evoked changes, not shown). Relative changes in sensory-evoked MUA rates were rather similar to the changes in spontaneous MUA. Cooling significantly decreased early sensory-evoked MUA rates in L4 and B5B/6A (*p* < 0.001, *n* = 22), and evoked heterogeneous changes in L5 (Figure 8C). We also found that the grouping index calculated for spontaneous MUA positively correlated with the early evoked MUA firing rate (Figure 8D): the layers with a high grouping index displayed higher firing rates during the early sensory-evoked response. In contrast to the early MUA response, cooling significantly decreased the late MUA rates through all cortical layers (Figure 8F). The depth profiles of relative MUA changes, similar to spontaneous activities, displayed a maximum in L5 and minimum in L4 and around B5B/6A (Figures 8C,F). Taken together, despite moderate cortical cooling increasing the duration of cortical active states in anesthetized rats, it also strongly inhibited sensory-evoked MUA response in L4 and at B5B/6A and, in particular, the late component of sensory-evoked MUA response though all cortical layers.

## Discussion

The main finding of the present study is that the effects of cortical cooling on electrical activity are complex and differ across cortical layers and that these changes closely match a transition from slow-wave activity towards desynchronized network activity. Cortical hypothermia promoted a transition from slow-wave activity to more continuous persistent activity, and this was associated with a decrease in neuronal firing in all cortical layers except L5. We found that the MUA grouping index decreased through all cortical layers with cooling. While cortical cooling increased total time spent in active states, sensory-evoked responses were suppressed during cooling through all layers. With cooling, spontaneous and sensory-evoked MUA rates were more strongly reduced in L4 and B5B/6A, which are the main recipient layers of the thalamic sensory inputs. The cooling-dependent slowdown was detected at the fast time-scale with a decreased frequency of the sensory-evoked HFO.

### Hypothermic State vs. Desynchronized State

We suppose that partial inactivation of potassium channels with moderate/light cooling is the main reason for changes in slow-wave network dynamics, leading to changes in firing rates as a network effect. The majority of open membrane channels at rest, which maintain the resting membrane potential and determines the neuronal input resistance, are potassium leak channels (Hodgkin and Huxley, 1952; Lesage, 2003). It had been shown that one of the candidates for potassium leak channels, the two-pore-domain potassium [K(2P)] channel, is temperature-sensitive (Talley et al., 2001; Kang et al., 2005; Schneider et al., 2014). Moderate cooling evoked K(2P)-dependent neuronal activation in hippocampal cell cultures (de la Peña et al., 2012). Mild/moderate (~5°C below normothermia) local cooling in songbirds slows down the song tempo (Long and Fee, 2008; Zhang et al., 2017), but did not prevent the behavior itself in contrast to deep cortical cooling, used for local cortical inactivation (Lomber et al., 1994).

In sharp contrast with intracellular recordings in cats where slow/delta waves is a feature of slow-wave sleep (Steriade et al., 2001; Timofeev et al., 2001), transitions between synchronization and desynchronization have been reported in the rodent barrel cortex also during quiet and alert-whisking wake states. In the absence of whisker movements, large-amplitude membrane potential fluctuations dominate in L2/3 pyramidal cells and interneurons, whereas during active whisking, the large-amplitude membrane potential fluctuations were replaced by smaller amplitude and continuous fluctuations (Crochet and Petersen, 2006; Gentet et al., 2010; Reimer et al., 2014). In the rat barrel cortex, desynchronization suppressed spontaneous spiking activity of L2/3 principal cells but caused enhanced firing in a subset of L5 neurons (de Kock and Sakmann, 2009). Application of cholinergic agonists to the thalamus in urethane-anesthetized mice also caused a desynchronized state in the barrel cortex and suppression of L2/3 principal neuron firing (Hirata and Castro-Alamancos, 2010), but in contrast to other desynchronization models (Gentet et al., 2010; Sakata and Harris, 2012), enhanced fast-spiking neuronal firing. In the rat visual cortex, basal forebrain stimulation decreased MUA firing rates in the superficial layers and lightly increased firing in other layers (Goard and Dan, 2009). In the rat auditory cortex, spontaneous desynchronization and desynchronization evoked by whisking in unanesthetized rats, and desynchronization evoked by electrical stimulation of the pedunculopontine tegmental nucleus in anesthetized rats, suppressed spontaneous spiking activity in the superficial layers but caused heterogeneous changes in deep layer firing. Typically, large tufted L5 pyramidal cells increased the firing rate (Sakata and Harris, 2012). The mean frequency of spontaneous excitatory postsynaptic potentials (EPSPs) is lower in superficial cortical layers in anesthetized rats (compared to deep layers), but the EPSP amplitude is higher, so, a single EPSP can evoke more than one action potential in the superficial layers (Zhu and Connors, 1999). Spontaneous superficial-layer spiking activity during active states is sparse (Brecht et al., 2003; Kerr et al., 2007; Poulet and Petersen, 2008; Sakata and Harris, 2009). In our experiments, cooling-evoked desynchronization led to largely consistent changes in spontaneous MUA firing rates across cortical layers including variable changes in L5 MUA frequency which might reflect a variable response of different L5 neurons and reduced firing in other cortical layers as it occurs during slow-wave—persistent network activity transitions. Analysis of the sensory-evoked response latency, amplitude and slope supported the idea that cortical cells were depolarized during cooling-evoked active states (Shu et al., 2003a; Crochet et al., 2005; Rosanova and Timofeev, 2005).

### MUA Detection and Cooling-Dependent Slowdown

To compare MUA frequency in control and during cortical cooling we corrected spike amplitudes for their decrease upon cooling. At the first approximation, the extracellular spike amplitude is proportional to the first derivative of the intracellular potential, diminished with cooling, because spikes become wider (Henze et al., 2000; Volgushev et al., 2000; Girardin and Martin, 2009). Using a constant amplitude threshold for MUA detection might lead to artificially decreased/increased firing rates with lower/higher temperatures, respectively. We discarded small-amplitude extracellular events from our analysis, but we cannot exclude that some portion of the detected MUA spikes were not somatic spikes, in particular, in L4 and B5B/6A. It has been reported that up to 20% percent of the early sensory-evoked MUA response in L4 and at the border between layers 5 and 6 are thalamocortical axonal spikes (Vinokurova et al., 2018). The early sensory-evoked MUA response, insensitive to the glutamate receptor antagonists, had been also reported in rat pups (Minlebaev et al., 2007).

We found that cortical cooling decreased the frequency of HFO and delta wave slopes, but did not change SEP slopes. Cooling did not change the depth profile calculated for LFP delta wave onset, but significantly changed the depth profile of LFP delta wave cessation. Interneurons are probably less sensitive to cooling compared to principal cells (Vizi and Sperlágh, 1999; Motamedi et al., 2013). Therefore, fast network dynamics, based on neuron-interneuron interactions, might be temperature-sensitive. Cortical feed-forward inhibition plays a central role in the generation of cortical gamma oscillations (Bartos et al., 2007; Whittington et al., 2011; Khazipov et al., 2013) that could explain the significant reduction in HFO frequency with cooling.

### Evoked and Spontaneous MUA During Cooling-Evoked Desynchronization

We found that the polarity of changes in spontaneous L5 MUA rates correlated with changes in the total time spent in the active state (negative correlation with total silent time) along with a decrease in slow-wave occurrence during cooling-evoked desynchronization. Layer 5 neurons, the largest neurons in the cortex (Feldman, 1984), play the central role in triggering and propagation of cortical active states (Sanchez-Vives and McCormick, 2000; Chauvette et al., 2010; Wester and Contreras, 2012; Beltramo et al., 2013). Since L5 neurons were able to generate prolonged active states during decreased activity in other layers, they probably also play a central role in the maintenance of persistent cortical activity (Shu et al., 2003b).

A decrease in slow-wave occurrence was associated with a decrease in the MUA grouping index through all cortical layers. During slow oscillations, neuronal membrane potential rapidly changes between the hyperpolarized and depolarized states with short time delays in neighboring neurons (Steriade et al., 1993, 2001; Chauvette et al., 2010; Sheroziya and Timofeev, 2014); this strongly increases the probability of firing together. The MUA grouping index (mean number of MUA spikes within the ±10 ms window around a spike) mainly provides an estimation of neuronal synchrony near the electrode (and therefore is proportional to local neuron density) with a possible contribution of intrinsically bursting cells and interneurons, whose ISIs can be less than 10 ms. The depth profile of mean MUA rate during the early sensory-evoked response also depends on local neuron density (and thalamic sensory inputs) explaining the similarity with the depth profile of the grouping index (Figure 8D).

The reduction in MUA rates with cooling might depend on intrinsic neuronal properties and slow-wave occurrence. L2/3, L4, and L5B in the rodent somatosensory cortex contain intrinsically bursting neurons (Chagnac-Amitai et al., 1990; Schubert et al., 2001; Staiger et al., 2004; de Kock and Sakmann, 2008; Prõnneke et al., 2020). First, intrinsically generated high firing rates require fast neuronal membrane re-polarization during the generation of the action potential. With cooling, the voltage-gated potassium current has a higher activation threshold and lower amplitude (Volgushev et al., 2000), which might decrease the frequency of spikes within bursts. Second, cortical neurons intrinsically generate bursts of spikes (or higher firing rates) right after fast depolarization from a hyperpolarized membrane potential with subsequent adaptation (Connors et al., 1982; Chagnac-Amitai et al., 1990; Connors and Gutnick, 1990; Llinás et al., 1991; Nuñez et al., 1993; Gray and McCormick, 1996; Brumberg et al., 2000; Contreras, 2004; Staiger et al., 2004; Hedrick and Waters, 2012). Thus, intrinsically bursting neurons can potentially decrease mean firing rates after the transition to desynchronization. Third, the relative fraction of interneurons across all cortical layers is around 14% (Sirota et al., 2008; Sakata and Harris, 2009; Reyes-Puerta et al., 2015), however, interneuron density was reported to be maximal in L2, L4, and L5 in the rat barrel cortex and the interneuron/principal neuron ratio in these layers is 0.19–0.25 (Meyer et al., 2011; Reyes-Puerta et al., 2015). Similarly to bursting neurons, interneurons can decrease firing after the transition to desynchronization (Gentet et al., 2010; Sakata and Harris, 2012). Nevertheless, the distribution of bursting neurons and interneurons through the barrel cortex does not strictly match L4 and B5B/6A and does not fully explain the differences between spontaneous and evoked MUA ratios.

We observed that MUA rates during the early response decreased in L4 and B5B/6A and then decreased through all cortical layers (late response); in other words, the response became shorter with cooling (Figures 7B, 8). On average, peak firing was not diminished with cooling. The L4 vs. L5 and L5 vs. B5B/6A contrast decreased in the late response compared to the early response. The sensory-evoked triphasic response is composed of fast initial excitation, prolonged (intracortical) inhibition, and then delayed excitation (Chapin et al., 1981; Zhu and Connors, 1999). A feature of the barrel cortex is fast VPM-dependent feed-forward inhibition lasting up to 10 ms after cortical activation (Agmon and Connors, 1992; Gil and Amitai, 1996; Gabernet et al., 2005; Sun et al., 2006; Cruikshank et al., 2007). Interneurons are probably less sensitive to cooling than principal cells (Vizi and Sperlágh, 1999; Motamedi et al., 2013). The decreased frequency (~445 Hz in control) and increased HFO peak power with cooling might indicate changes in neuron-interneuron interactions during thalamus-dependent feed-forward inhibition. It was reported that in regular-spiking cells the mean latency from onset of excitation to the onset of the inhibitory outward current was about 2.4 ms (~420 Hz) during disynaptic inhibition in L4 (Cruikshank et al., 2010). Therefore, we propose that hypothermia changed the excitation/inhibition balance (Higley and Contreras, 2006) towards interneurons during the sensory-evoked response. Spiny stellate cells and fast-spiking interneurons form clusters of highly interconnected cells in L4 (Feldmeyer, 2012; Koelbl et al., 2015). In mice, L6A also contains clusters of cells called infrabarrels (Crandall et al., 2017) and has a local inhibitory circuit (Frandolig et al., 2019). From a statistical point of view, the connectivity of local inhibitory cells (and overall inhibitory impact on MUA) might depend on local neuron density that has local maxima in L4 and B5B/6A. Also, it had been reported that tonic inhibition (Semyanov et al., 2004), mediated by extrasynaptic GABA(A) receptors, decreases with cooling (Bright and Smart, 2013), while activation of tonic inhibition in L4 (targeting fast-spiking interneurons) reduces thalamus-dependent feed-forward inhibition (Swadlow, 2003; Krook-Magnuson et al., 2008). Thus, cooling could provide a strong inhibitory effect on MUA in L4 and B5B/6A during the early response. The instantaneous amount of non-spiking cells (decreased MUA rate) was higher during hypothermia that could increase peak firing (Figure 8A) in the early response (Reig et al., 2015).

The thalamocortical circuit should be viewed as a single functional and dynamic entity (Crunelli and Hughes, 2010; Sheroziya and Timofeev, 2014) and thalamic contribution to cortical slow oscillations had been already shown (David et al., 2013; Lemieux et al., 2014). Neuronal activity in L4 and B5B/6A in the barrel cortex might strongly depend on the thalamus because cells within these cortical layers receive weak synaptic inputs from other cortical layers (compared to thalamocortical). Spiny stellate cells in L4 do not have excitatory inputs from other cortical layers in the home column while L4 star pyramids have weak and sparse synaptic inputs (Feldmeyer, 2012). Brief high-frequency stimulation of thalamocortical axons in slices triggers widespread recurrent activity in populations of neurons in L4 and then spreads into the adjacent L2/3 and L5 (Beierlein et al., 2002). In our experiments, cooling-evoked cortical desynchronization could decrease the occurrence of thalamic spontaneous low-threshold calcium bursts (Llinás and Yarom, 1981; Timofeev and Steriade, 1996) and respectively decrease spontaneous firing (Figure 6) in L4 and B5B/6A (Swadlow and Gusev, 2001). A cortical MUA response to a spontaneous thalamic activation could decrease similarly to the changes in the sensory-evoked MUA response during hypothermia, but the relative thalamic/intracortical impact on spontaneous MUA rates in L4 and B5B/6A remained unclear. Further investigations are required to verify thalamic contribution to vertical propagation of activity at the beginning of delta waves in the barrel cortex.

## Data Availability Statement

The raw data supporting the conclusions of this article will be made available by the authors, without undue reservation.

## Ethics Statement

The animal study was reviewed and approved by the Institutional Animal Care and Use Committee of Kazan State Medical University N9-2013.

## Author Contributions

GB, KC, and MS performed the experiments. MS and RK designed the research project and wrote the article. MS analyzed the data.

## Conflict of Interest

The authors declare that the research was conducted in the absence of any commercial or financial relationships that could be construed as a potential conflict of interest.
